# Research on the Cutting Force and Serrated Chips in Ultra-Precision Micro-Grooving of SLM Ti6Al4V Alloy

**DOI:** 10.3390/mi14030533

**Published:** 2023-02-24

**Authors:** Zhongpeng Zheng, Jiajing Guo, Ruilin Gao, Xin Jin

**Affiliations:** 1 Department of Mechanical Engineering, Tsinghua University, Beijing 100084, China; 2 Department of Mechanical Engineering, Beijing Institute of Technology, Beijing 100081, China

**Keywords:** serrated chip, micro-grooving, cutting force, SLM Ti6Al4V alloy

## Abstract

Selective laser melting (SLM) has significant advantages in the near net shape manufacturing of metal parts with complex geometries. However, SLM parts usually have problems such as poor surface quality and low dimensional accuracy, which require post-processing. This paper focuses on the research around the influence of ultra-precision micro-grooving the SLM Ti6Al4V alloy on the cutting force and serrated chips. The influence of the processing parameters on the cutting force and surface processing quality was analyzed in detail, and the cutting simulation model of the SLM Ti6Al4V alloy was established. The formation process of the serrated chip was successfully simulated, and the experiments verified the reliability of the established model. The research results show that the dynamic cutting force and surface processing quality are mainly related to the depth of cut, and the two trends are consistent. It is also shown that the serrated chip begins on the free surface of the workpiece and propagates deeply in the shear zone, forming a shear band, and its serrated nodules move upward and forward to form periodic serrated chips.

## 1. Introduction

Additive manufacturing (AM) technology has significant advantages in the rapid prototyping and near net line manufacturing of complex-shaped parts and has great potential in aerospace, biomedical and other fields [1,2,3]. Therefore, additional post-processing for current AM parts is required to improve the dimensional machining accuracy and surface quality. At present, selective laser melting (SLM) is one of the world’s most widely used additive manufacturing technologies. It is being actively used in different metal materials [4], especially Ti6Al4V, which is lightweight and high strength with excellent properties such as corrosion resistance and fatigue resistance. Therefore, the SLM process benefits the fabrication of Ti6Al4V near net-shaped complex parts, significantly reducing material waste and the consequent manufacturing cost of expensive materials [5,6]. However, compared to traditional manufacturing techniques, the surface quality and dimensional accuracy of additively manufactured parts are generally lower due to layer-by-layer processing and step effects. This is a key problem that additive manufacturing needs to solve, and these problems limit SLM [7]. Therefore, post-processing of the SLM Ti6Al4V alloy is required.

In recent years, relevant scholars have conducted processing research on the post-processing of additive manufacturing [8,9,10,11]. Ni et al. studied the effect of material anisotropy on the machinability of the SLM Ti6Al4V alloys [12]. Campos et al. performed a comprehensive micro-milling machinability analysis comparing standard Ti6Al4V produced using SLM and Ti6Al4V in terms of the cutting force, specific cutting force, surface roughness, top burr height and chip morphology [13]. Bai et al. studied the influence on the machinability of ASTM A131 steel manufactured using AM in post-milling processing [14]. Lizzul et al. carried out slot milling on the additively manufactured Ti6Al4V titanium alloy with different cutting parameters to evaluate the effect of the stacking direction on the surface quality, focusing on the surface topography. The results showed that the skewness parameter could distinguish the machined surface of AM parts in different directions [15]. Airao et al. studied the machinability of titanium alloys fabricated using AM and traditional material. These results show that SLM Ti6Al4V produces high tool wear and poor surface quality [16].

In addition, SLM Ti6Al4V has a low thermal conductivity and a relatively slow heat dissipation. It is relatively easy to form adiabatic shear bands and serrated chips in traditional cutting, which accelerates the tool wear and affects the surface quality of the machined parts. To reduce the experimental cost and time constraints, it is necessary to conduct a finite element numerical analysis to overcome the experimental limitations and better explain the necessary methods of the experimental mechanism [17]. P. Didier et al. performed end milling tests on Ti6Al4V parts produced using SLM additive manufacturing and verified the consistency of the cutting force signals between the experiments and the simulation [18].

Although much research has been done on traditional titanium alloy processing, there needs to be more research on the machinability of the SLM Ti6Al4V alloys, especially the finite element modeling analysis of the SLM Ti6Al4V alloys in serrated chips and the cutting force. Therefore, this paper investigates the serrated chip formation process of additively manufactured parts and the influence of the machining quality during micro-grooving.

## 2. Materials and Method

### 2.1. Experiment Setup and Materials

**Figure 1:**
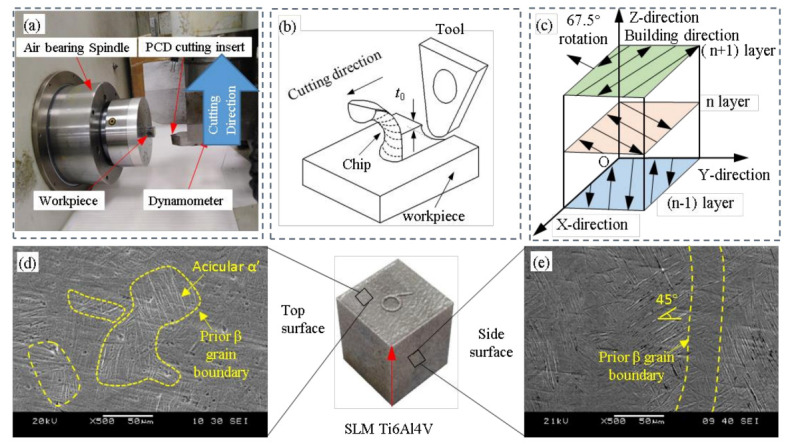
(**a**) Experimental setup; (**b**) schematic of the processing process; (**c**) fabrication strategy of SLM Ti6Al4V; (**d**,**e**) microstructure of SLM Ti6Al4V.

Figure 1 is a schematic diagram of the experimental processing device and the parameters of the fabrication strategy for SLM Ti6Al4V. In this study, the micro-grooving experiments were carried out on an ultra-precision diamond lathe ULG100. The experimental setup is shown in Figure 1a,b. A Kistler 9251C1 dynamometer and a 5051A amplifier were used in the experiments, and the cutting forces were recorded at a sampling rate of 1 kHz. The workpiece was SLM Ti6Al4V and the tool material was a polycrystalline diamond tool (PCD). The tool arc radius, rake angle and relief angle were 0.4 mm, 0° and 7°, respectively. Before micro-grooving, the surface of the workpiece was ground first to ensure the flatness of the sample surface and its stability during cutting. The detailed experimental processing parameters and tool parameters are shown in Table 1.

The experimentally processed sample SLM Ti6Al4V was prepared using a 67.5° scanning strategy. The laser power was 200 W, the spot size was 75 μm, the thickness of each layer was 30 μm, the laser scanning speed was 1500 mm/s and the laser scanning spacing was 65 μm. The detailed preparation strategy is shown in Figure 1c. Before preparing SLM Ti6Al4V, Ti6Al4V was preheated to approx. 80 °C and argon was flushed into the forming chamber to maintain the inert gas to prevent the oxidation of SLM Ti6Al4V. In this paper, the growth direction of the SLM Ti6Al4V sample is defined as the Z direction, the plane XOY is the top surface, the plane XOZ direction is the side surface and the plane YOZ direction is the front surface. The prepared sample size was 12 × 12 × 15 mm.

The material’s microstructure directly determines the material’s mechanical properties and affects the cutting force. Since the SLM Ti6Al4V alloy has a line-by-line layered stacking structure, the sample microstructures in each direction are different. Figure 1d,e shows the microstructure of the top and side of the SLM Ti6Al4V alloy under the 67.5-degree scanning strategy, and the results show that the microstructures of the two different surfaces are completely different. There are a large number of columnar grains on the side of the sample. These columnar grains are formed by the epitaxy of prior β grains passing through multiple deposition layers along the direction of the heat flow. There are a large number of acicular martensite inside the columnar grains. However, the prior β grains at the top of the sample are irregular polygons and belong to the basket structure. These different microstructures also mean that SLM Ti6Al4V differs from traditional processing.

After the sample was processed, a JEOL JSM-5500 scanning electron microscope (SEM) was used to observe the chip morphology. A Veeco3D confocal microscope was used to observe the profile of the processed surface to evaluate the surface processing quality. In addition, for the reliability of the FEM model established in this paper, the accuracy of the FEM was verified by comparing the geometric characteristics and the cutting force of the chips in the experimental and simulation processes as the evaluation criteria. In this section, the chips generated under different cutting parameters were collected and mounted using epoxy resin, and after polishing and etching, the chip morphology was obtained using scanning electron microscopy (SEM).

### 2.2. Numerical Simulation

**Figure 2:**
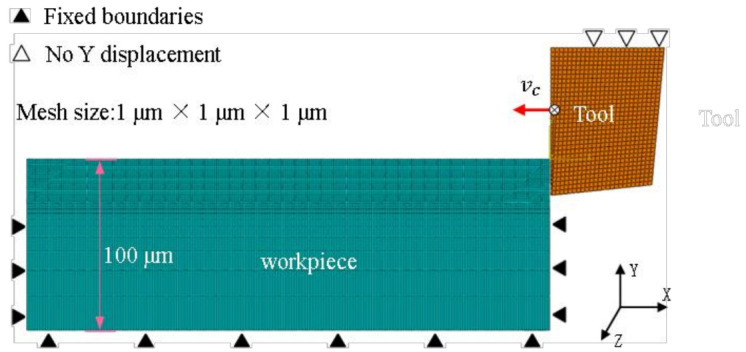
Finite element cutting simulation modeling.

In this section, Abaqus/Explicit was used to model the cutting process model of SLM Ti6Al4V, and the cutting model was divided into meshes using Lagrangian meshing technology. As shown in Figure 2, the workpiece in the cutting model was divided into two parts. The upper part of the chip layer was a grid with a size of 1 μm × 1 μm × 1 μm and the farther away from the cutting layer, the sparser the grid became, improving the efficiency. The material properties of the processed workpieces were the same. The tool’s rake angle α of the tool was 0° and the relief angle was 6°. The machine tool was set as a rigid body without deformation. During the cutting process, the bottom and sides of the workpiece were fixed, and the tool had only the degree of freedom in the X direction. At the same time, the tool’s rake face was defined as the main contact surface and the workpiece was defined as the slave contact surface. In addition, the physical and mechanical properties of Ti6Al4V used in the cutting simulation model are shown in Table 2, and the physical and mechanical properties of the tool are shown in Table 3. It should be pointed out that the FEM model established did not consider the mechanical anisotropy of the material. These related research methods can also be found in the research of other scholars [19]. Although the material’s mechanical properties were different, it did not affect the serrated chip of the formation mechanism.

Johnson and Cook first proposed the Johnson–Cook model, which has been widely adopted in metal cutting simulations for considering the effects of strain hardening, strain rate hardening and thermal softening. At the same time, the model reflects the mechanical stress of metals under high strain, a high strain rate and high temperature, and is usually used for ductile materials. The flow stress is expressed by Equation (1) [23].

(1) $$\sigma =\left(A+B\varepsilon_{p}^{n}\right)\left(1+C\ln\frac{\dot{\varepsilon }}{{\dot{\varepsilon }}_{0}}\right)\left[1-{\left(\frac{T-T_{r}}{T_{m}-T_{r}}\right)}^{m}\right]$$

where 𝜀_*p*_, $\dot{\varepsilon }$ and ${\dot{\varepsilon }}_{0}$ are the equivalent plastic strain, equivalent plastic strain rate and reference strain rate, respectively. *T_m_* and *T_r_* represent the melting temperature and room temperature, respectively. A, B, C, *n* and *m* represent the initial yield stress, hardening modulus, strain rate sensitivity coefficient, strain rate dependency coefficient and thermal softening coefficient, respectively. The detailed parameters of the JC constitutive are shown in Table 4.

In order to simulate the failure model of the material, the paper adopted the JC damage fracture model, and the specific formula is as follows [25]

(2) $${\bar{\varepsilon }}_{\mathrm{JC}}=\left[D_{1}{+D}_{2}\exp\left(D_{3}\frac{P}{\bar{\sigma }}\right)\right]\left[{1+D}_{4}\ln\left(\frac{\dot{\varepsilon }}{{\dot{\varepsilon }}_{0}}\right)\right]\left({1+D}_{5}\left(\frac{{T\;-\;T}_{r}}{T_{m}{\;-\;T}_{r}}\right)\right)$$

where ${\bar{\varepsilon }}_{\mathrm{JC}}$ denotes the critical equivalent plastic strain, the ratio of $P/\bar{\sigma }$ is defined as the stress triaxiality and $D_{1}$, $D_{2}$, $D_{3}$, $D_{4}$ and $D_{5}$ denote the damage parameters of the material. The detailed parameters of the JC damage model are shown in Table 5.

## 3. Results and Discussion

### 3.1. Surface Morphology of SLM Ti6Al4V

**Figure 3:**
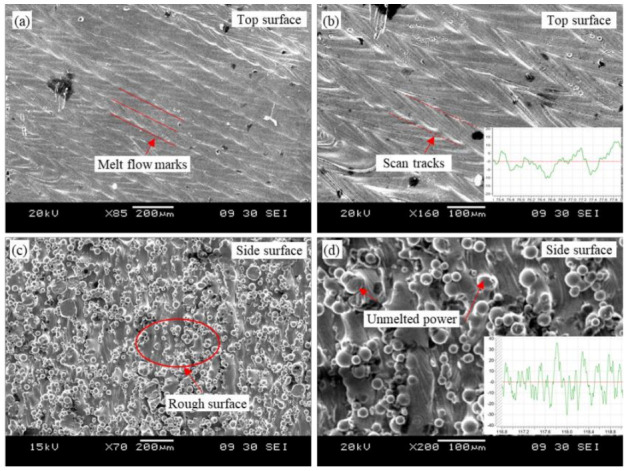
Surface morphology of SLM Ti6Al4V. (**a**,**b**) top surface. (**c**,**d**) side surface.

Surface topography is an important characteristic that can be used to evaluate the surface quality of SLM parts. However, since the additive manufacturing process is a top-down, layer-by-layer processing method, the environment and temperature fields on the sides of the sample are different during the manufacturing process. The scanning traces and unmelted particles adhering to the sample also affect surface roughness. Figure 3 shows the surface morphology of SLM Ti6Al4V. From Figure 3a,b, it can be found that there were obvious traces left by the laser scanning on the top of the sample, and the surface topography clearly showed the laser scanning track during the SLM process. In addition, some impurities were present on the sample surface, which may be partly due to the particles blown into the build area by the aeration unit of the SLM machine before the top layer was fully cured [27]. It can be found in Figure 3c,d that there were obvious unmelted particles on the side of the sample sticking to the surface of the workpiece, and these powder particles reduced the overall surface quality of the SLM part. Therefore, SLM Ti6Al4V required post-processing to meet the requirements of the high-precision parts.

### 3.2. Cutting Force

The cutting force is an important factor in evaluating the machinability of materials, and it is closely related to the materials’ mechanical properties and processing parameters. Compared to the traditional Ti6Al4V alloy, the hardness, yield strength and ultimate tensile strength of SLM Ti6Al4V were significantly higher than that of the forged Ti6Al4V alloy, which also meant that the cutting force was different from the traditional titanium alloy, which had specific research significance [19].

**Figure 4:**
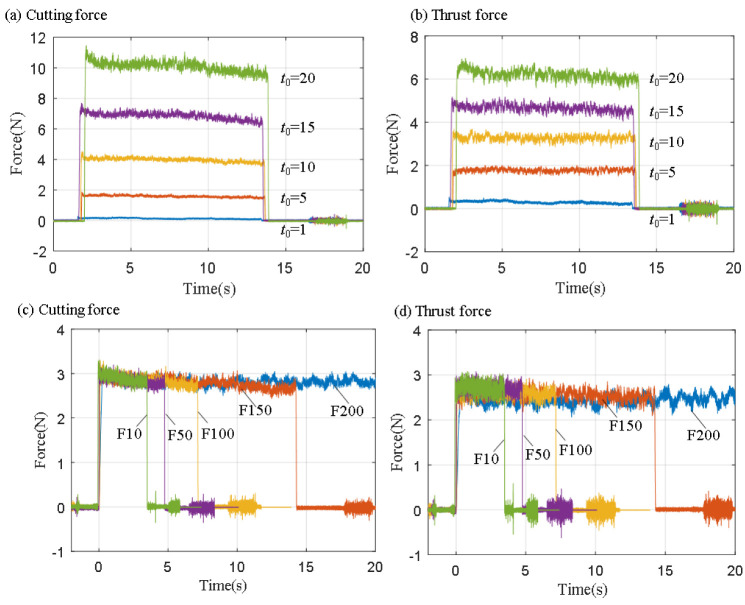
Dynamic cutting force curve. (**a**,**b**) the cutting speed was 60 mm/s, with different depths of cut; (**c**,**d**) the depth of cut was 8 μm, with different cutting speeds.

During ultra-precision machining, the cutting force is closely related to the mechanical properties and microstructure of the material. Figure 4 shows the dynamic cutting force curves under the different cutting process parameters. Figure 4a,b shows that, when the cutting speed was 60 mm/min, the cutting force increased gradually with the increase in the cutting parameters. Moreover, with the same parameters, the cutting force received by the tool is generally greater than the thrust. However, it should be noted that when the depth of cut is smaller than the radius of the blunt circle of the tool edge, for example, when the depth of cut was 5 μm, due to the size effect, all the thrust of the tool is greater than the cutting force. Figure 4c,d shows the effect of the cutting speed on the cutting force. It can be observed from the figure that when the fixed depth of cut was 8 μm, the cutting force and thrust of the tool did not change significantly after the cutting speed was gradually increased from 10 mm/min to 200 mm/min. The result means that the cutting speed had a negligible effect on the cutting forces. In addition, the dynamic cutting force spectrum in Figure 4 shows an obvious drastic phenomenon, which was believed to be the result of a serrated chip formation, and found that the greater the depth of cut, the greater the fluctuation of the cutting force [28].

**Figure 5:**
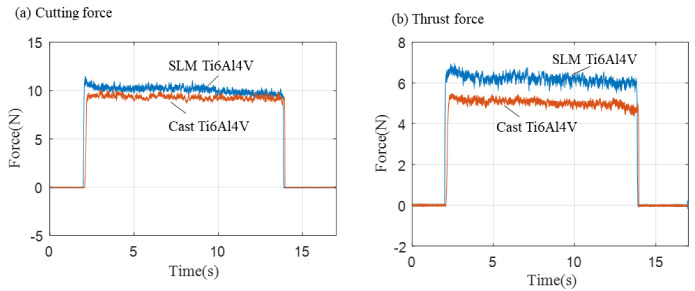
Comparison of the dynamic cutting force curve between SLM Ti6Al4V and cast Ti6Al4V, the depth of cut was 20 μm, and the cutting speed was 60 mm/s. (**a**) cutting force; (**b**) thrust force.

In order to further understand the difference between the titanium alloy and the traditional cast titanium alloy, it was necessary to compare the differences in the cutting force between them. Figure 5 shows the cutting force comparison results of traditional cast Ti6Al4V. The results show that the cutting force of the additively manufactured titanium alloys was slightly larger than that of traditional cast materials. This was mainly attributed to the higher yield strength of the SLM titanium alloys than conventional materials [29].

### 3.3. Surface Topography

**Figure 6:**
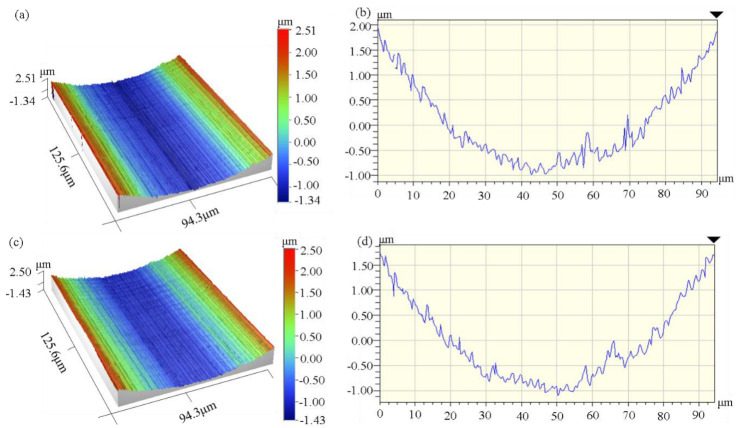
Morphology and profile of the micro-grooved surface for the speed corresponding to 60 mm/min; (**a**,**b**) the depth of cut was 5 μm; (**c**,**d**) the depth of cut was 15 μm.

Figure 6 shows the topography of the micro-grooves processed using ultra-precision machining SLM Ti6Al4V, and it can be found that the processed micro-grooves had a good profile. Owing to the consistent tool parameters, the micro-grooves bottom profile was constant.

**Figure 7:**
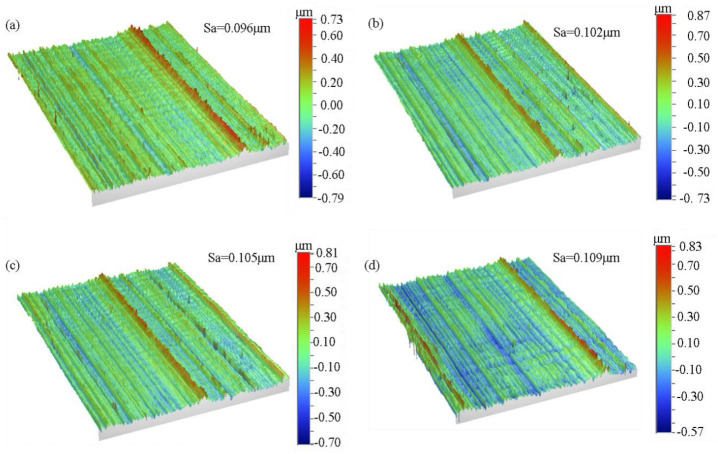
Roughness measuring the surface for the depth of cut corresponding to 8 μm. (**a**) 50 mm/min; (**b**) 100 mm/min; (**c**) 150 mm/min: (**d**) 200 mm/min.

**Figure 8:**
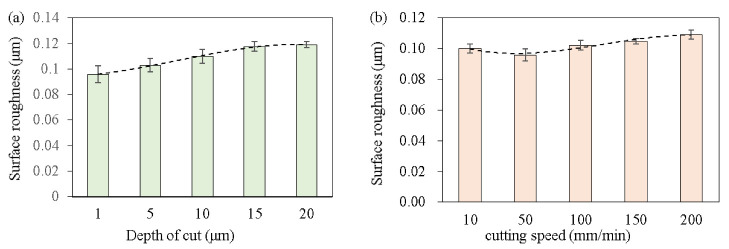
Roughness results of the micro-groove machined surface; (**a**) the cutting speed was 60 mm/s, (**b**) the depth of cut was 8 μm.

The roughness is an important index to evaluate the processing quality of the micro-groove. In this paper, the surface roughness (Sa) was used to evaluate the quality of the processed surface. In addition, to evaluate the profile roughness of the micro-grooves and avoid the errors caused by the grooves and inclinations, the sample profile should be flattened (cylinder and tilt) before the roughness evaluation. Figure 7 shows the actual rough surface of the sample processed at different cutting speeds, and the texture along the cutting direction can be seen. Figure 8 shows the experimental results of the roughness. When the constant cutting speed was 60 mm/min, the surface roughness (Sa) gradually increased with the increase in the depth of cut, which was consistent with the results of the dynamic cutting force. However, when the constant depth of cut was 8 μm, the surface roughness (Sa) did not increase with the increase in the cutting speed. The surface roughness fluctuated around 0.100 μm, which means that the cutting speed has little effect on the surface processing quality.

### 3.4. Serrated Chip and FE Analysis

**Figure 9:**
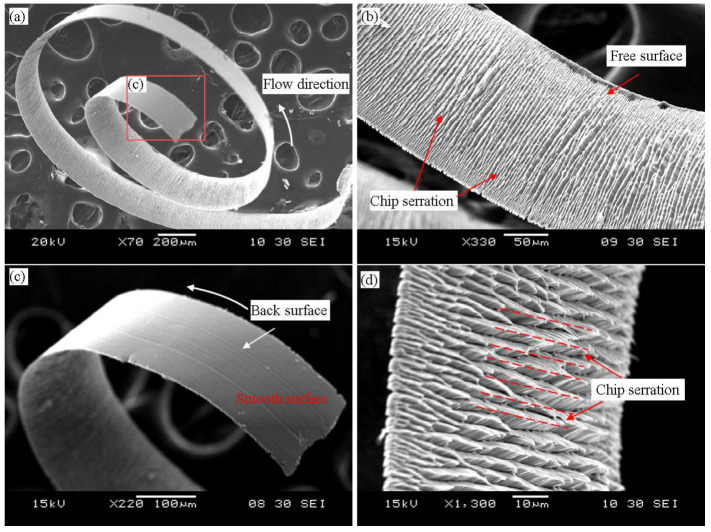
Chip morphology of micro-grooving experiments for the speed corresponding to 60 mm/min, and the depth of cut was 10 μm. (**a**) overall chip morphology; (**b**,**d**) free surface of the chip; (**c**) back surface of the chip.

**Figure 10:**
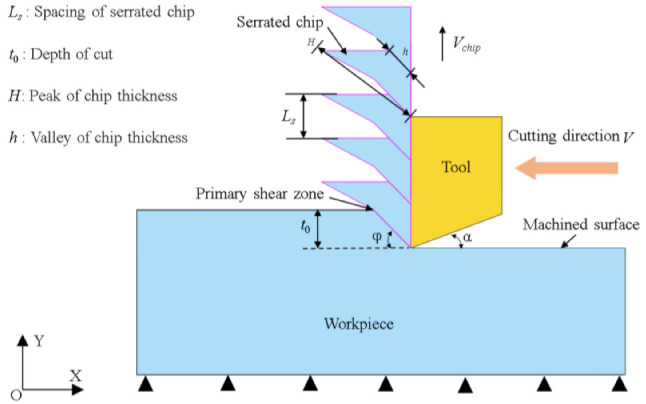
Schematic diagram of the serrated chip formation process.

The chips in ultra-precision machining are a critical evaluation of machining performance. The state of the chips can provide important information during the cutting process. In addition, the cutting force and surface roughness are also affected by chips [30]. Figure 9 shows the micro-grooving chip morphology diagram of the ultra-precision machining SLM Ti6Al4V. During the cutting process of SLM Ti6Al4V, the chips were affected by the sliding friction between the tool’s rake face and the back of the chip, plastic deformation and cutting heat, which caused the chips to produce certain regular sawtooth chips. Figure 9a shows the chip completion process. As shown in Figure 9b,d, periodic serrations appeared in the chips due to local shearing, which indicated the formation of shear bands. The specific formation process and geometry of the serrated chips are shown in Figure 10.

**Figure 11:**
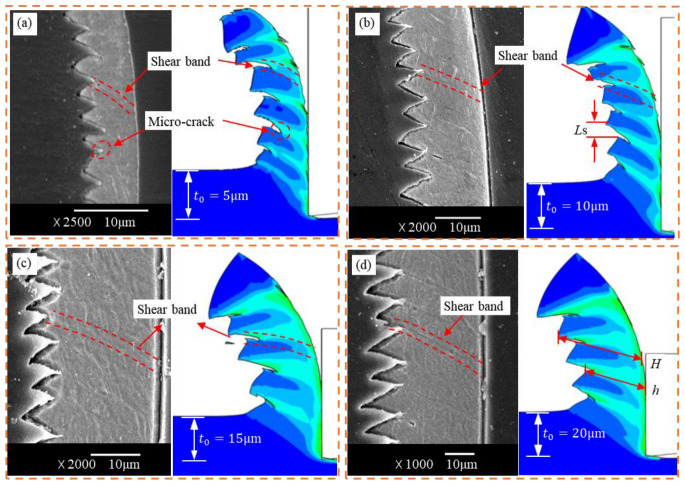
Comparative results of the simulated and experimental serrated chips for a cutting speed of 60 mm/min; (**a**) t_0_ = 5 μm, (**b**) t_0_ = 10 μm, (**c**) t_0_ = 15 μm, (**d**) t_0_ = 20 μm.

To further quantify the formation law of the serrated chips and verify the accuracy of the finite element model, the chip samples were prepared and polished to obtain a cross-sectional view of the chips. Due to the serrated chip morphology and cutting force being closely related to the field variables (stress, strain, temperature), the chip geometrical characteristics and cutting forces were generally employed as an evaluation criterion to verify the accuracy of the FEM [28]. The chips generated under different cutting parameters were collected and inlaid using epoxy resins. After polishing and etching, the chip morphology was obtained using scanning electron microscope (SEM). Figure 11 compares the serrated chip morphology generated by the SLM Ti6Al4V cutting experiment. The simulated chip morphology correlated significantly with the experimental serrated chip. In addition, periodic adiabatic shear bands and micro-cracks were observed in the serrated chips. The serrated chips were micro-cracks on the chip-free surface caused by material extrusion [31], which induced the formation of shear bands. During micro-grooving, the reduction in the load carrying capacity of the material caused the shear band workpiece to move upward and forward in the nodules, eventually forming a complete serrated chip.

**Figure 12:**
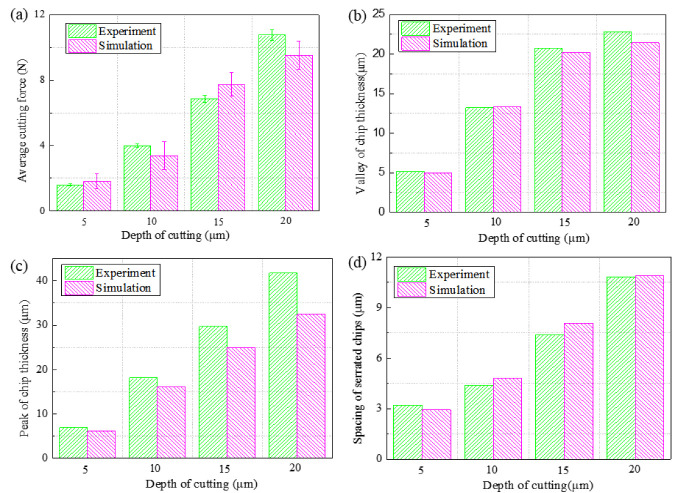
Comparison of the experimental and simulation results at different depths of cut; (**a**) average cutting force, (**b**) valley of chip thickness, (**c**) peak of chip thickness, (**d**) spacing of serrated chips.

Figure 12 compares the cutting forces, valley and peak of the chip thickness, valley and spacing of the serrated chips. Figure 12a presents the cutting force of the experimental result with the simulated cutting force. Irrespective of the experimental or simulation results, the cutting force increased with increases in the depth of cut, and the cutting force error was within 6.5%. Figure 12b,d compares the geometric characteristics of the experimental and simulated serrated chip. The relative errors between the experimental and simulated results concerning the valley of the chip thickness, the peak of the chip thickness and the spacing of the serrated chips were between 2.3% and 20%. The observed differences at different cutting parameters were satisfactory and acceptable, which inversely verified the reliability of the established finite element simulation model. Due to a material error and uniformity modeling results, there was a certain deviation between the actual simulation results and the experiment, and the maximum deviation was about 20%.

## 4. Conclusions

The selective laser melting (SLM) process has significant advantages in the near net shape manufacturing of metal parts with complex geometries. Unfortunately, SLM parts usually suffer from poor surface quality and low dimensional accuracy, requiring post-processing. Therefore, this paper focused on the influence of ultra-precision micro-grooving of the SLM Ti6Al4V alloy on the cutting force and serrated chip, established a finite element cutting simulation model of the SLM Ti6Al4V alloy and verified the reliability of the established model through experiments. The specific research is as follows.

(1) The cutting simulation prediction model of the SLM Ti6Al4V alloy was established and the cutting force and serrated chip parameters predicted under the same cutting parameters agreed with the experimental results. The research results showed that the serrated chip began on the free surface of the workpiece and propagated deeply in the shear zone to form a shear band. The nodules moved upward and forward, forming the serrated chips.
(2) The dynamic cutting force was mainly related to the depth of cut and gradually increased. The dynamic cutting force gradually increased and the range was between 0–10 N. However, the cutting speed parameters had less influence on the dynamic cutting forces.
(3) The surface roughness (Sa) of ultra-precision micro-grooving of the SLM Ti6Al4V alloy fluctuated around 0.1 μm and its Sa gradually increased with the increase in the depth of cut. However, the correlation with the cutting speed was small and its changing trend was consistent with the cutting force.

## Figures and Tables

**Table 1:** Details of the workpiece parameters and machining parameters.

Workpiece	Material	SLM Ti6Al4V, Cast Ti6Al4V
Tool parameters	Material Rake angle (α) Clearance angle $\mathrm{Nose}\;\mathrm{radius}\;(\;R_{n})$ $\mathrm{Edge}\;\mathrm{radius}\;(\;R_{e})$	polycrystalline diamond (PCD) 0° 7° 0.4 mm ~1 μm
Trimming parameters	Depth of cut Spindle speed Feed speed	2 μm 600 rpm 1.5 mm/min
Cutting parameters	$\mathrm{Depth}\;\mathrm{of}\;\mathrm{cut},\;t_{0}$ Cutting speed, v	1, 5, 10, 15 and 20 μm 60 mm/min
	$\mathrm{Depth}\;\mathrm{of}\;\mathrm{cut},\;t_{0}$ Cutting speed, v	8 μm 10, 50, 100, 150, 200 mm/min

**Table 2:** The physical and mechanical properties of the workpieces [20,21].

Density (kg/m^3^)	Elastic Model (GPa)	Poisson’s Ratio	Heat Conduction (W/M°C)	Specific Heat Capacity (J/Kg°C)	Thermal Expansion Coefficient (/°C)	Melting Temperature (°C)
4430	109	0.34	6.8	611	9.0 × 10^−6^	1605

**Table 3:** Physical parameters of the cemented carbide cutting tools [22].

Density (kg/m^3^)	Elastic Model (GPa)	Poisson’s Ratio	Heat Conduction (W/m K)	Specific Heat Capacity (J/kg K)
119,000	534	0.22	50	400

**Table 4:** Johnson–Cook constitutive model parameters for Ti6Al4V [24].

A (MPa)	B (MPa)	C	m	n
997.9	653	0.0198	0.7	0.45

**Table 5:** Johnson–Cook damage model parameters for Ti6Al4V [26].

$D_{1}$	$D_{2}$	$D_{3}$	$D_{4}$	$D_{5}$
−0.09	0.25	−0.5	0.014	3.87

## Data Availability

The data is contained within the article.
